# Extracellular Heme Proteins Influence Bovine Myosatellite Cell Proliferation and the Color of Cell-Based Meat

**DOI:** 10.3390/foods8100521

**Published:** 2019-10-21

**Authors:** Robin Simsa, John Yuen, Andrew Stout, Natalie Rubio, Per Fogelstrand, David L. Kaplan

**Affiliations:** 1 Department of Biomedical Engineering, Tufts University, Medford, MA 02155, USA; robin.simsa@verigraft.com (R.S.);; 2 VERIGRAFT AB, 41346 Gothenburg, Sweden; 3 Wallenberg Laboratory, University of Gothenburg, 41345 Gothenburg, Sweden; Per.Fogelstrand@wlab.gu.se

**Keywords:** cell-based meat, cultured meat, skeletal muscle tissue engineering, muscle constructs, bioartificial muscle, heme proteins, hemoglobin, myoglobin, meat color, tissue color, bovine myosatellite cells, cellular agriculture

## Abstract

Skeletal muscle-tissue engineering can be applied to produce cell-based meat for human consumption, but growth parameters need to be optimized for efficient production and similarity to traditional meat. The addition of heme proteins to plant-based meat alternatives was recently shown to increase meat-like flavor and natural color. To evaluate whether heme proteins also have a positive effect on cell-based meat production, bovine muscle satellite cells (BSCs) were grown in the presence of hemoglobin (Hb) or myoglobin (Mb) for up to nine days in a fibrin hydrogel along 3D-printed anchor-point constructs to generate bioartificial muscles (BAMs). The influence of heme proteins on cell proliferation, tissue development, and tissue color was analyzed. We found that the proliferation and metabolic activity of BSCs was significantly increased when Mb was added, while Hb had no, or a slightly negative, effect. Hb and, in particular, Mb application led to a very similar color of BAMs compared to cooked beef, which was not noticeable in groups without added heme proteins. Taken together, these results indicate a potential benefit of adding Mb to cell culture media for increased proliferation and adding Mb or Hb for the coloration of cell-based meat.

## 1. Introduction

Muscle tissue engineering in vitro may provide new treatments for skeletal muscle diseases such as muscular dystrophies or trauma. Another application of muscle tissue engineering is the generation of meat, derived from livestock animal cells, for human consumption, here referred to as cell-based meat (other common names include “cultured meat”, “in vitro meat”, or “cellular agriculture”). The rationale for developing cell-based meat is the potential to decrease resource intensity and increase environmental sustainability of meat production [1] compared to current industrial animal farming, which is associated with issues of greenhouse gas emission, land usage [2], deforestation [3], biodiversity [4], antibiotic resistance [5], and animal welfare. The ability to grow meat in defined bioreactor conditions also potentially allows a decrease in the use of steroid hormones [6] and antibiotics [7], while increasing the content of health-related proteins and vitamins by defined nutrient composition of cell culture media. Currently, the generation of muscle tissue in large quantities is not cost-efficient, since knowledge about muscle tissue engineering was generated mainly related to medical applications. Therefore, more basic research regarding optimization and production of muscle tissue for food products is necessary.

The main component of meat is skeletal muscle cells, which, therefore, is a logical starting cell type for cell-based meat production. However, differentiated skeletal muscle cells cannot be expanded in vitro, as they lose their ability to proliferate in normal conditions [8,9]. In contrast, myosatellite cells, which are muscle progenitor cells, have a greater capacity to proliferate and differentiate [10]. Myosatellite cells can be harvested from non-lethal biopsies and then grown in 3D to form bioartificial muscle (BAM) constructs by growth in hydrogels, on synthetic or natural scaffolds or on native decellularized extracellular matrix [11,12,13]. Differentiation of myosatellite cells into adult muscle tissue can be initiated by changing the media conditions, topographical cues, electrical or mechanical stimuli, or other modes [14]. Three-dimensional growth of skeletal muscle in hydrogels along agarose pillars was the basis of the first demonstration of a cell-based meat prototype [15,16]. Growth of skeletal muscle tissue requires the presence of functional proteins, which are commonly added with fetal bovine serum or by the addition of specific growth factors (e.g., fibroblast growth factor (FGF) or vascular endothelial growth factor (VEGF)) [17]. Other functional proteins which could have an important role in cell-based meat development are heme proteins, such as myoglobin (Mb) or hemoglobin (Hb). 

Heme proteins contain a prosthetic group with a bound iron atom and fulfill functions such as oxygen binding and transport to mitochondria, oxidative phosphorylation, and intracellular catalysis [18,19]. Hb is primarily found in red blood cells, while Mb is found in native skeletal and cardiac muscle tissue [20]. The content and redox form of Mb is the main contributor to the color of meat [21] and is associated with the typical bloody, metallic taste of meat [22]. These attributes are of great interest for cell-based meat development, as both taste and color of meat are crucial for consumer acceptance [23]. Skeletal muscle cells only produce limited amounts of Mb in vitro when proliferating, and Mb content in vitro is lower compared to in vivo muscle tissue [24]. Therefore, increasing the content of heme proteins during muscle tissue formation may lead to a more meat-like composition and appearance. Heme proteins such as Hb are already widely used in meat products, as natural color enhancers, binders, or fat replacers [25]. Also, the addition of heme proteins to food products can help to reduce iron deficiency, which affects up to 20% of the world population [26].

Increasing heme protein expression in skeletal muscle cell culture may be achieved by adaption of growth conditions, such as hypoxic incubation or addition of certain supplements, but raises issues in terms of food regulatory aspects, production feasibility and/or cell viability. For example, muscle cells are one of the few cell types that proliferate well in hypoxic conditions [27], limiting coculture systems. Another option of increasing the heme protein content is the addition of extracellular heme proteins in growth media directly during cell proliferation and differentiation, which can have a positive effect on cell metabolism [19,28,29]. Recently, the commercially available meat substitute “Impossible Burger” has incorporated heme proteins originating from soy (leghemoglobin) into a soy-based alternative for ground beef, which led to a more meat-like taste compared to a control group without added heme [30]. These results showed the potential of heme proteins to be used in meat alternatives to recreate natural meat taste and flavor. However, the incorporation of heme proteins in cell culture for cell-based meat production has not yet been investigated.

The aim of this study was to investigate the effects of direct additions of the heme proteins Hb and Mb on skeletal muscle cell proliferation, differentiation, coloration, biochemical activity, and viability. Experiments were performed on primary isolated bovine satellite cells (BSCs). For tissue formation, cells were cultured in a fibrin hydrogel and differentiated along 3D printed anchor point constructs. Color measurements and heme content were compared to commercially available ground beef.

## 2. Materials and Methods

### 2.1. Bovine Satellite Cell Isolation and Cell Culture

Primary bovine satellite cells were isolated from the semitendinosus of a 60-day-old male Charolaise x Simmental beef cow raised at the Tufts Cummings School of Veterinary Medicine. Briefly, a small excision (~0.5 cm^3^) was taken according to methods approved by the Tufts University Institutional Animal Care and Use Committee (IACUC Protocol #G2018-36), placed in dulbecco’s modified eagle medium (DMEM) + Glutamax (ThermoFisher, Pittsburg, PA, USA) with 1% antibiotic–antimycotic, and transported to the lab on ice, where fat and connective tissue were removed before the remaining muscle was minced into a thick paste. Minced tissue was divided into 50 mL tubes with 20 mL DMEM Glutamax and centrifuged at 200× *g* for 5 min. The media was aspirated, and the tissue was resuspended in 10 mL DMEM + Glutamax with 0.2% collagenase II (Worthington Biochemical, Lakewood, NJ, USA; 275 U/mg). This digestion solution was incubated for 45 min at 37 °C, with micropipette triturations performed every 15 min. Then, the solution was triturated using an 18-gauge blunt-tipped needle until it passed through the needle easily, incubated at 37 °C for another 15 min, and again triturated several times with an 18-gauge needle. Next, 20 mL of growth media comprised of DMEM + Glutamax supplemented with 20% FBS, 1 ng/mL human FGF-basic (100-18B, PeproTech, Rocky Hill, NJ, USA), and 1% Primocin (Invivogen, San Diego, CA, USA) was added to both tubes to halt digestion. Digests were filtered through 70 μm and 40 μm cell strainers, centrifuged at 200 g for 5 min, and resuspended in growth media. Cells were then counted on a hemocytometer, plated at a density of 100,000 cells/cm^2^ onto uncoated T75 tissue-culture flasks, and incubated in a 37 °C with 5% CO_2_. After 24 h, the media was collected from flasks to separate slowly adherent satellite cells from quickly adherent fibroblasts and transferred directly to new tissue-culture flasks coated with 1 μg/cm^2^ mouse laminin (Millipore, Burlington, MA, USA). Flasks were left untouched for three days, at which point growth media was changed, and cells were cultured using standard practices on tissue-culture plastic coated with iMatrix recombinant laminin-511 (NC1547124, iMatrix-511, Fisher, Waltham, MA, USA). After two weeks of culture, puromycin in growth media was replaced with 1% antibiotic–antimycotic. For differentiation, media comprised DMEM + GlutaMax enriched with 2% FBS and 1% Antibiotic–Antimycotic solution.

### 2.2. Proliferation Assay

A proliferation assay was performed using CyQuant Reagent (ThermoFisher), following the supplier’s instructions. Briefly, BSCs were seeded in a 96-well plate at a density of 500 cells/well. Cell culture media consisted of either standard proliferation media or proliferation media with added hemoglobin from bovine blood (Sigma, St Louis, MO, USA) or myoglobin from equine skeletal muscle (Sigma) in concentrations of 1, 3, or 5 mg/mL. Both proteins were provided by the supplier in the oxidized met redox form (metmyoglobin or methemoglobin). Four plates were prepared with 6 replicates per group (*n* = 6) and single plates were recovered after 1, 3, 5, and 7 days of incubation by aspirating the media and storage at −80 °C. 100 µL media was aspirated and replaced by fresh media after 4 days. When all plates were recovered, CyQuant working solution was prepared by diluting the supplied lysis reagent 1:20 in sterile H_2_O, followed by the addition of the dye reagent to a dilution of 1:400. Plates were thawed at room temperature, and 200 µL of CyQuant working solution was added to each well. Fluorescence was measured at an excitation of 480 nm and emission of 520 nm with a spectrophotometer (Synergy H1, Biotek, Winooski, VT, USA). Cell number was calculated with a standard curve of cells seeded at a known density.

### 2.3. 3D BAM Formation

BSC differentiation was based on anchor point attachment in a fibrin hydrogel. To allow elongation and differentiation of BSCs along 2 anchor points, an anchor point construct fitting into individual wells of a 24-well plate in different confirmations was designed with SolidWorks and 3D printed on a desktop 3D printer (3DWOX 201, Sindoh, South Korea) with standard polylactide (PLA) filament (S1 File). One day prior to BSC seeding, individual wells of 24-well plates were treated at RT with 1 mL of 5% Pluronic F-127 (P2443, Sigma) to decrease cell attachment to the well surfaces. After 30 min, the liquid was aspirated and the plate was kept with open lid in the flow hood for 1–2 h to ensure complete evaporation. Anchor point constructs were sterilized with 70% ethanol and stored together with the 24-well plate overnight under UV light.

For BAM formation, BSCs at a density of 3.5 × 10^6^ cells/well were combined with 20 mM CaCl_2_ in proliferation media. For groups with heme protein, proliferation media also contained Hb or Mb at a concentration of 3 mg/mL. Cell mixture was added to individual wells with pre-added thrombin from bovine plasma (Sigma) with a total enzyme activity of 0.6 U. Finally, bovine concentrated plasma fibrinogen stock (341573, EMD Millipore, Bedford, MA, USA) diluted in H_2_O was added to make up a final concentration of 3 mg/mL, and the solution was mixed quickly by pipetting up and down multiple times. All reagents were sterile-filtered prior to use. The total volume of the cell-reagent mix was 800 µL. Furthermore, the fibrinolytic inhibitor aminocaproic acid (ACA) (Sigma), at a concentration of 1 mg/mL, was added to BSC-Fibroin mix, as well as to media, to prevent rapid hydrogel degradation. To observe muscle tissue formation without fibrinolysis inhibitor, one batch was incubated without ACA. Plates were incubated for at least 30 min, at 37 °C, to initiate hydrogel polymerization. Then, 1.2 mL of respective proliferation media containing either no or 3 mg/mL of heme proteins was added to each well, and incubation continued at 37 °C. After 1 day, media was aspirated and replaced with differentiation media. Every 3 days, media was replaced with fresh media, containing the respective heme proteins. After 9 days, BAMs were removed with forceps from anchor-point construct for further analysis. As a control for hydrogel compaction, gel without added cells was used. Prior to tissue harvesting, thickness and width were measured with a digital caliper while still on the construct. The length was equal to spacing between the 3D constructs´ anchor points (13.14 mm end-to-end). Weight was measured with a standard lab balance. For further analysis, groups were compared against commercially available ground beef (80% lean, 20% fat) purchased from a local supermarket.

### 2.4. Time-Lapse Imaging

Initial BAM formation was visualized with a BZ-X710 fluorescent microscope (Keyence, Japan). Then, 3.5 × 10^6^ BSCs in a fibrin hydrogel were prepared, as described above, and incubated for 30 min at 37 °C, to induce gelation. Media was then added, and the plate was transferred to the microscope in a thermo-, and gas-stable chamber at 37 °C and CO_2_ of 5%. Images of different regions of the gel were taken with z-Stack every 15 min for 12 h. Time-lapse video was prepared with KEYANCE BZ-X Analyzer software at 2 frames per second.

### 2.5. Biochemical Analysis

Nitric oxide release of BAM was quantified with a commercially available kit (Total Nitric Oxide and Nitrate/Nitrite Parameter Assay Kit, R&D Systems, Minneapolis, MN, USA), by following the supplier’s instructions. Briefly, BAMs were grown in fibrin hydrogel with differentiation media, and media recovered after 72 h of incubation (*n* = 5). The media was centrifuged for 3 × 10 min at 13,400 rpm to remove particles, and 50 of µL supernatant (diluted 1:5) was added to a 96-microwell plate. Indirect measurement of nitric oxide was performed by measuring the level of nitric oxide metabolites nitrate and nitrite assay with Griess reagent, and absorbance measured at 540 nm. Concentration was calculated from a nitrite and nitrate standard. Soluble Glycosaminoglycan (GAG) released by the cells was measured with a GAG Kit (Chondrex, Redmond, WA, USA). Briefly, media was removed from BAMs, following 72 h of incubation, and added to a 96-well plate in duplicates (*n* = 6) with different dilutions. Samples were then either incubated in 100 µL of Dye Reagent or 100 µL of PBS as a control, and absorbance was measured at 525 nm. Concentration was calculated from a known standard.

### 2.6. DNA Quantification

DNA from 7–15 mg of wet tissue was extracted using the DNeasy Blood and Tissue Kit (Qiagen, Hilden, Germany), following the manufacturer’s instructions. Briefly, BAM samples (*n* = 4) were incubated at 55 °C with proteinase K solution, until the tissue was completely digested. DNA was extracted with spin columns and measured with a Qubit 3.0 Fluorometer (ThermoFisher). The total amount of DNA was calculated from a known DNA standard.

### 2.7. Immunohistochemistry

To verify the identity and myogenicity of isolated bovine satellite cells, they were cultured in 2D and stained for Pax7 during proliferation, or for Troponin T, following one week of differentiation. To evaluate cell development in fibrin hydrogels, BAMs were cultured for 9 days, and stained for Troponin T. For all immunofluorescent staining, the same protocol was used. Briefly, cells or BAMs were fixed at room temperature for 30 minutes using 4% paraformaldehyde, washed in PBS 3×, and stored in PBS at 4 °C before staining. For staining, cells were permeabilized for 15 minutes using 0.5% Triton-X (Sigma, St Louis, MO, USA) in PBS, blocked for 45 minutes, using 5% goat serum (Gibco) in PBS with 0.05% sodium azide (Sigma), and washed 3x with PBS containing 0.1% Tween-20 (Sigma). Primary Pax7 antibodies (Thermo Fisher, #PA5-68506) were diluted 1:100 in blocking solution containing 1:100 Phalloidin 594 (Thermo Fisher, Pittsburg, PA, USA; #A12381) and added to 2D proliferating cells. Primary Troponin T antibodies (developmental studies hybridoma bank, CT3) were diluted to 4 μg/mL in blocking solution containing 1:100 Phalloidin 594 (Thermo Fisher, #A12381) and added to 2D differentiated cells or BAMs. Primary antibodies were incubated overnight at 4 °C. The following day, cells or BAMs were washed 3× with PBS + Tween-20. For 2D, secondary antibodies for Pax7 (Invitrogen, Carlsbad, CA, USA; goat-anti-rabbit AlexaFluor-488, #A-11008, 1:500) and Troponin T (Thermo Fisher Scientific goat-anti-mouse AlexaFluor-488, #A-11001, 1:1000) were diluted in blocking solution and added to cells for 1 h at room temperature. Cells were washed 3× with PBS + Tween-20 and mounted with Fluoroshield mounting medium with DAPI (Abcam, Cambridge, UK) before imaging. For BAMs, secondary antibodies for Troponin T were diluted in blocking solution containing DAPI (Thermo Fisher, #62248, 1:1000) and added to constructs for 1 h at room temperature. Constructs were washed 3× and stored in PBS + Tween-20 for imaging.

### 2.8. Live–Dead Staining and Alignment Quantification

After 8 days of incubation, BAMs were incubated with differentiation media containing 2 µM calcein AM and 4 µM of ethidium homodimer (L3224, ThermoFisher) for 30 min at 37 °C. Live–dead stained samples (*n* = 3) were then Z-stack and/or XY-stitch imaged, using the 488 nm and 594 nm filters on a BZ-X700 fluorescent microscope (Keyence, Japan). The alignment of muscle cells was quantified from calcein AM images by ImageJ with the Fiji plugin. Briefly, images were transformed into 16-bit type and analyzed individually with the “Directionality” tool. The analysis was set to Fourier components, 90 bins and −90° to +90°. The obtained histogram was normalized to the main axis, e.g., the orientation angle with most alignment. Normalized data were used for calculation of aligned structures within 10° and −10° of the main axis.

### 2.9. Scanning Electron Microscopy (SEM)

BAMs were dehydrated for 10 minutes at a time in 35%, 60%, 80%, 90%, 95%, and 100% ethanol, after which they were transferred into hexamethyldisilazane (Sigma) for 10 min. Hexamethyldisilazane was then aspirated and samples were left to air-dry in a fume hood. Prior to SEM imaging, samples (*n* = 5) were sputter-coated with gold for 120 s, using a SC7620 sputter coater (Quorum Technologies, East Sussex, UK). Imaging was performed on a Zeiss EVO-10MA scanning electron microscope (Zeiss, Jena, Germany).

### 2.10. Color Image Analysis

BAMs were placed, while still on the 3D-printed anchor construct, on a petri dish with a white background, within a flow hood, and images were taken with a digital camera (Canon DS126311), with 2 fluorescent lamps as illumination. Camera settings were ISO 200 and 1/20 s exposure. Images of beef samples were also taken in the same conditions. Images were then analyzed on ImageJ (v1.51, NIH) for RGB colors, by measuring several areas per sample and calculating the mean. RGB colors were furthermore converted into L*a*b* colors, where L* shows lightness, a* red/green spectra, and b* blue/yellow spectra of the sample. Overall changes of color compared to either fresh beef or cooked beef was expressed as delta E (ΔE), following the CIE76 formula:

(1) $$\Delta E=\sqrt{{\left({L^{*}}_{2}-{L^{*}}_{1}\right)}^{2}+{\left({a^{*}}_{2}-{a^{*}}_{1}\right)}^{2}+{\left({b^{*}}_{2}-{b^{*}}_{1}\right)}^{2}},$$

where L*_2_, a*_2_, and b*_2_ express the values of the respective BAM sample, while L*_1_, a*_1_, and b*_1_ express the average values of either fresh or cooked beef. ΔE expresses the relative color difference between 2 samples. While ΔE = 0 shows identical colors, low values indicate high similarity, and high values indicate low similarity between two colors.

### 2.11. Cooking of Beef Samples

To compare color of BAMs with cooked beef, beef samples were cooked on a hot plate in a metal mold. First, beef samples were cut into small pieces, similar to the size of the BAMs. Prior to cooking, the weight of samples was measured and the hot plate heated to 145 °C. A stainless steel base mold (Thermo Fisher) was moistened with a small volume of olive oil and put in the center of the hot plate. After several minutes, a single sample was added onto the metal mold with forceps and heated for 30 s. Then, the sample was turned to the other side, and heating continued for 30 s. Finally, the sample was recovered and weighed after cooking. Then, images of the sample were taken for color analysis, as described above.

### 2.12. Total Pigment Extraction

Total pigment extraction was performed following previously published protocols [31,32]. Briefly, 20–60 mg samples of BAM and beef were added to a 0.1 M sodium phosphate buffer with a total volume of 10× the tissue weight and homogenized (Polytron PT 10-35, FisherSci, Hampton, NH, USA). Pigments were extracted for at least 1 h at RT, after which homogenate was centrifuged 3 × 15 min at 13,000 rpm, and the supernatant was taken for absorbance spectra measurement. Total pigments were calculated from a prepared standard of known concentration of Hb or Mb. BSCs, BSCs + Mb, and ground beef showed maximum absorbance at 409 nm (Metmyoglobin), BSCs + Hb showed maximum absorbance at 406 nm (Methemoglobin). Therefore, the concentration of different groups was calculated with the standard curve of the respective protein.

### 2.13. Statistical Analysis

GraphPad Prism 8 was used for all statistical analyses. For comparisons of multiple groups for only one parameter, one-way ANOVA and Tukey´s multiple comparison tests were applied. Significance is indicated in bar graphs with asterisk signs (*), indicating *p*-value of *p* ≤ 0.05 (*), *p* ≤ 0.01 (**), or *p* ≤ 0.001 (***). No significance (n.s.) is displayed for *p* > 0.05.

## 3. Results

### 3.1. Bovine Myosatellite Cell Characterization

**Figure 1:**
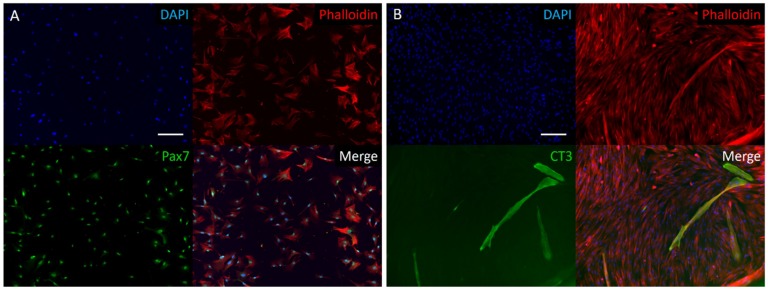
Two-dimensional immunofluorescence stain of isolated bovine muscle satellite cells (BSCs). (**A**) Proliferating bovine satellite stained for DAPI, actin cytoskeleton (Phalloidin), and Pax7, a nuclear marker of satellite cells. Stains show a highly pure satellite cell population, following isolation and pre-plating protocol. (**B**) Following one week of differentiation, cells were stained for DAPI, actin cytoskeleton (Phalloidin), and Troponin T (CT3), a marker of myogenesis. Scale bars are 200 µm.

Following a bovine muscle biopsy and cell expansion in vitro, characterization of proliferative cells by immunohistochemistry showed positive staining for the transcription factor Pax7, a nuclear identifier of myosatellite cell phenotype (Figure 1). Expression of Pax7 in all imaged cells indicates successful isolation of satellite cells from other cell types (i.e., fibroblasts), using the described pre-plating isolation method. After cultured cells reached confluency, they were differentiated for one week and stained for Troponin T. Expression of Troponin T, a sarcomeric protein involved in the contractile complex of skeletal muscle, indicates the myogenicity of isolated cells and verifies the successful isolation of a skeletal muscle precursor cell population.

### 3.2. Myoglobin Increases Proliferation of BSCs

**Figure 2:**
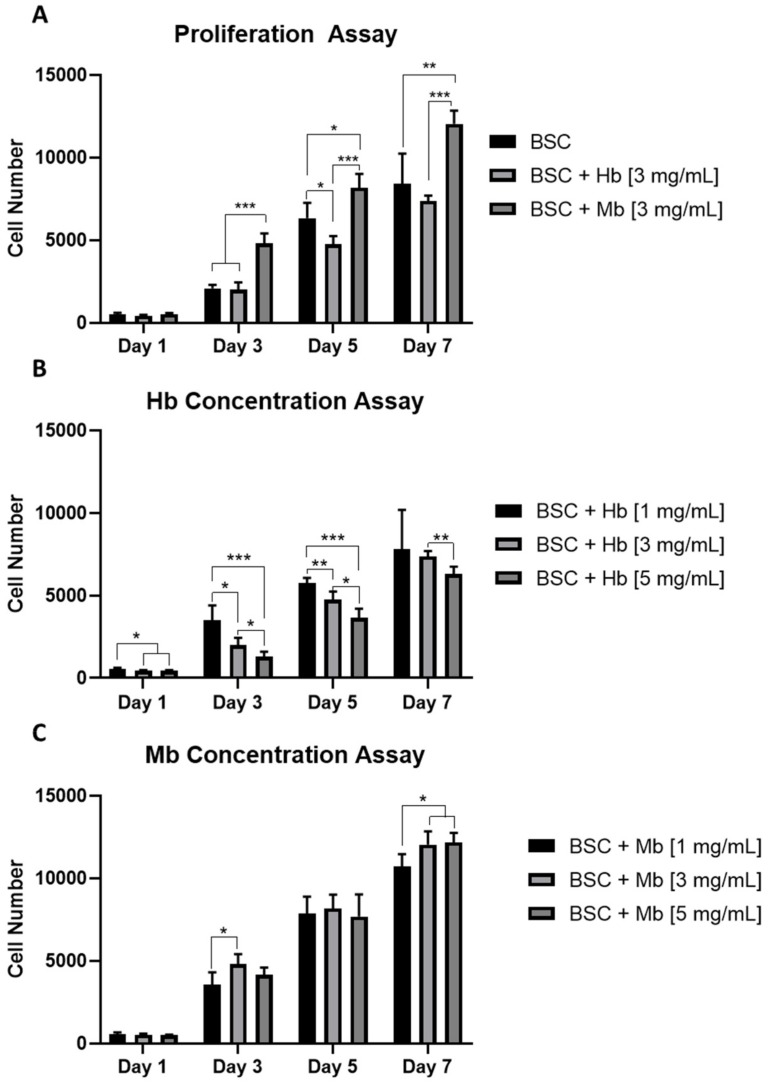
Proliferation of BSCs grown in the presence of different Hb or Mb concentrations in 2D. (**A**) Cell number of BSCs, BSCs + 3 mg/mL Hb, or BSCs + 3 mg/mL Mb, quantified with CyQuant reagent (*n* = 6) after one, three, five, and seven days. BSCs proliferation with different concentrations of (**B**) Hb and (**C**) Mb, at 1, 3 or 5 mg/mL was observed (*n* = 6). Cell number was calculated from a standard curve prepared from cells seeded at known density. * *p* ≤ 0.05, ** *p* ≤ 0.01, *** *p* ≤ 0.001.

The influence of Mb and Hb on the proliferation capacity of BSCs was observed by growing cells for one week with or without added heme proteins at different concentrations. From day three onward, Mb administered at 3 mg/mL resulted in a significant increase in proliferation compared to the untreated BSC group (Figure 2A). The Hb-treated group showed significantly decreased proliferation compared to the control group on day five and significantly decreased proliferation compared to the Mb group from day three on. Cell-doubling time in hours (calculated from initial cell number and cell number at day 7) was 41.67 ± 2.55 for BSCs, 43.28 ± 0.66 for BSC + Hb, and 36.63 ± 0.74 for BSC + Mb.

Additionally, the effects of the concentration of heme protein on the proliferation potential of BSCs were analyzed. A linear decrease in proliferation was observed in the Hb group from 1 mg/mL to 3mg/mL and 5 mg/mL, after one, three, and five days (Figure 2B). After seven days, significant differences only persisted between 3 mg/mL and 5 mg/mL. The protein concentration of Mb had less effect on cell proliferation; however, on day seven, protein concentrations of 3 mg/mL and 5 mg/mL showed a significantly higher cell number compared to 1 mg/mL (Figure 2C). Taken together, these results indicate that addition of Mb to the culture media increases the proliferation capacity of BSCs, while addition of Hb has either no or a slightly negative effect on proliferation. Furthermore, proliferation effects were concentration dependent for Hb, but less so for Mb.

### 3.3. BAM Formation and Dimensions

**Figure 3:**
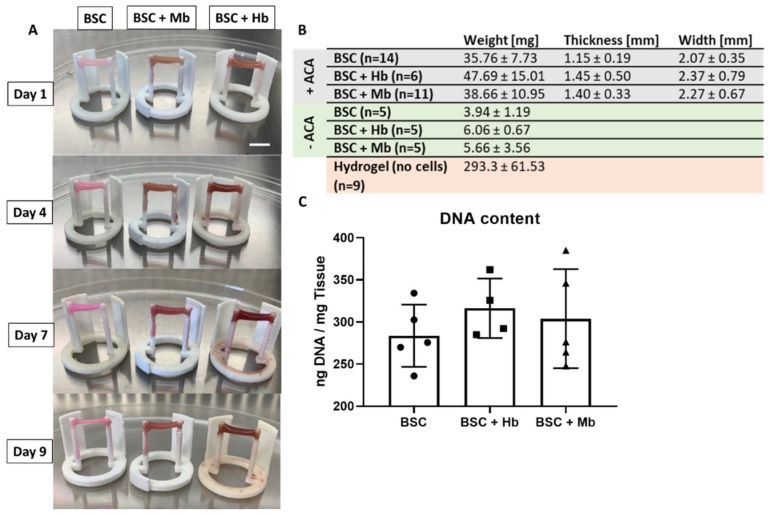
Properties of skeletal muscle tissue formation. (**A**) Representative images of bioartificial muscles (BAMs) generated from BSCs, BSCs + Hb, and BSCs + Mb (3 mg/mL for both heme proteins) at different time points (one, four, seven, and nine days of incubation), showing increased color intensity in constructs with Hb and Mb. Scale bar is 10 mm. (**B**) Weight, width, and thickness of BAMs at time point of harvest are presented. Groups were compared against groups without added ACA (–ACA) and fibrin gel without added cells, both incubated for an equal amount of time, in the same conditions, for comparison of hydrogel compaction by the cells. Width and thickness could only be measured in the +ACA BAMs, as –ACA BAMs detached from anchor points and lost their physical form. (**C**) DNA of BSCs, BSCs + Mb (*n* = 5), and BSCs + Hb (*n* = 4) was extracted by proteinase K degradation, and absorbance was measured with NanoDrop.

Previous studies have utilized Velcro glued to the opposite side of culture dishes or well-plates to serve as “anchor points” for muscle cells [33,34]; however, this procedure is operator-dependent, work-intensive and difficult to standardize. For this study, anchor-point constructs were designed and 3D-printed in different configurations (for 3D models, see S1 File) to fit into individual wells of a 24-well plate. BSCs were incubated for nine days in a fibrin hydrogel in order to allow maturation and elongation to a BAM along the anchor point axis, and to observe coloration effects of heme proteins at a concentration of 3 mg/mL (Figure 3A). Elongation and compaction of the BAMs along the anchor points were already observed within the first few hours, reaching a stable form after 10 h, both at the center (Video S1) and at the anchor points (Video S2) of the BAMs. To prevent fast degradation of the fibrin hydrogel by the cells, leading to detachment of BAMs from one or both anchor points, the fibrinolysis inhibitor aminocaproic acid (ACA) was added. A control group was grown, without ACA, to observe the effects of full hydrogel compaction. BAMs with ACA did not significantly differ in weight, thickness, or width (Figure 3B). When fibrinolysis was not inhibited (–ACA), the BAM weight was reduced by over 85% in all groups, showing that the hydrogel makes up a large part of the total muscle construct weight. Moreover, BAMs grown without ACA detached from the anchor point construct after 3–4 days of incubation. Even in the presence of fibrinolysis inhibitors (+ACA), muscle tissue weight was 80%–85% lower than the weight of the hydrogel without added cells, indicating partial gel compaction by the cells. Quantification of DNA content of the +ACA groups did not show significant differences among the groups (Figure 3C). 

### 3.4. BAM Morphology, Differentiation, and Viability

**Figure 4:**
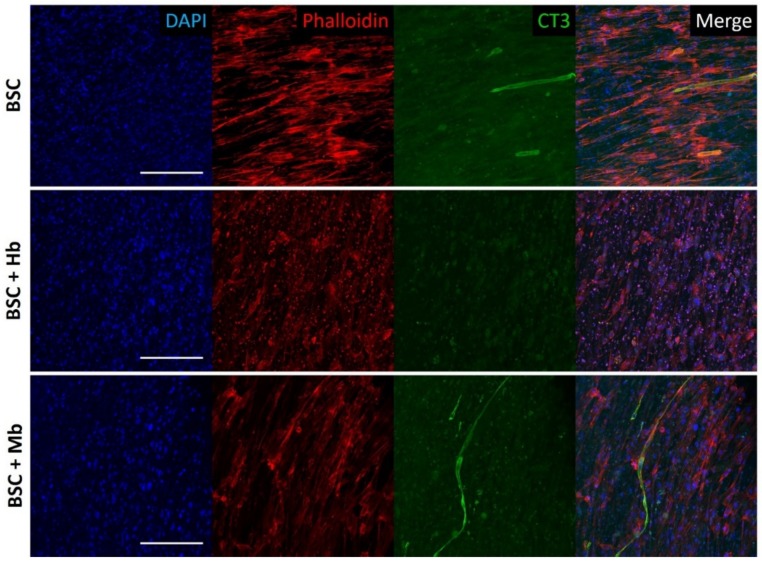
Confocal immunofluorescent imaging of BAMs. BAMs generated from BSC, BSC + Hb, or BSC + Mb (3 mg/mL for both heme proteins) were stained after eight days of differentiation for DAPI, actin cytoskeleton (Phalloidin), and Troponin T (CT3), a marker of myogenesis. Images show multinucleated myotube formation in BSC and BSC + Mb constructs, though not in BSC + Hb constructs. Scale bars are 200 µm.

Differentiation of BSCs into mature muscle cells in fibrin constructs was visualized through staining for Troponin T. In the BSC and BSC + Mb group, early differentiation after eight days was observed as indicated by positive Troponin T staining of long, tube-like multi-nucleated cells (Figure 4). These myotubes were aligned, following the general alignment of all cells, as indicated by Phalloidin staining of the actin cytoskeleton. While both these groups showed several elongated cells that were positive for Troponin T, the degree of myogenesis was moderate, and many cells did not appear to have formed myotubes. No major difference was seen in myogenicity between BSCs alone and BSCs treated with Mb. In contrast, the BSC + Hb group showed no positive staining for Troponin T, indicating little or no myogenic differentiation. At the same time, cells in these constructs appeared less elongated and aligned, indicating that cellular morphology in general was possibly adversely affected by the addition of Hb. Hence, both the BSC and BSC + Mb group showed early myogenesis, which was not observed in the BSC + Hb group.

**Figure 5:**
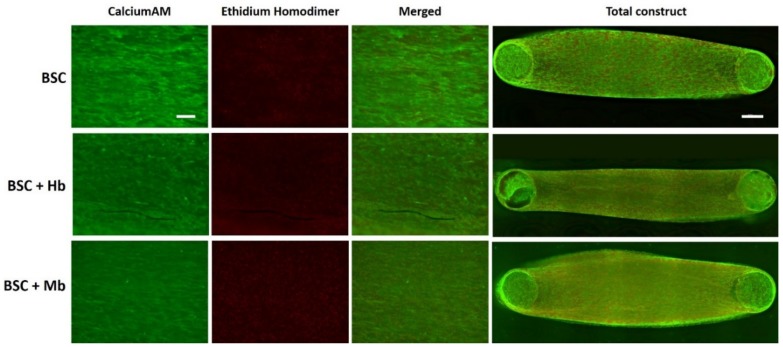
Live–dead staining of muscle constructs. BAMs generated from BSC, BSC + Hb, or BSC + Mb (3 mg/mL for both heme proteins) were stained with calcein AM (live cells, green) and ethidium homodimer (dead cells, red), to observe overall cell viability. Images were taken in green channel (488 nm) and red channel (594 nm), under identical microscope conditions, with z-Stack. For visualization of overall cell viability in total construct, x-y-stitching was performed. Scale bar in single channel images is 200 µm, and scale bar in the total construct is 1000 µm.

To visualize and compare cell viability among the different groups, BAMs were live–dead stained after eight days of incubation (Figure 5). The staining revealed no observable difference of live and dead cells among the different groups, suggesting that the addition of Hb and Mb does not impact cell viability at a visually detectable level. Green staining from calcein AM revealed a high number of viable cells in all groups, as well as elongated muscle-like morphologies and alignment along the anchor points. Red staining from ethidium homodimer indicated the presence of dead cells, particularly in the deeper layers of the tissue.

**Figure 6:**
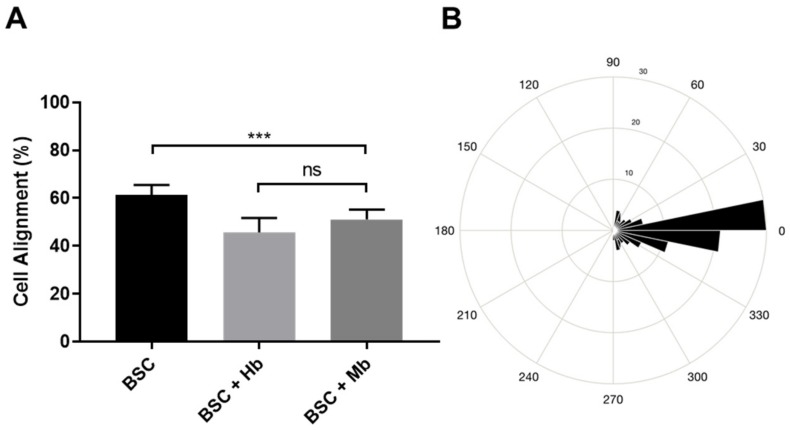
Alignment of BSCs. (**A**) BAMs generated from BSC, BSC + Hb, or BSC + Mb (3 mg/mL for both heme proteins) were stained with calcein AM and imaged with a fluorescent microscope, after which images were processed on Fiji with the Directionality tool (Fourier method). Cell alignment was determined as the percentage of structures that were aligned between −10° and 10° in relation to the axis of alignment (0°). Statistical significance was determined by one-way ANOVA and Tukey’s multiple comparison post hoc test (α= 0.05). Error bars are standard deviations (*n* = 9). (**B**) Average myotube orientation of the control group (BSC) was visualized by representative MatLab rose plot. *** *p* ≤ 0.001.

From the calcein AM staining, cell alignment was quantified. We found that cell alignment was highest in the BSC group (61.2%), significantly higher compared to the BSC + Hb (45.7%, *p* < 0.0001) and the BSC + Mb group (51.1%, *p* = 0.0005) (Figure 6), while significant, overall differences were small.

**Figure 7:**
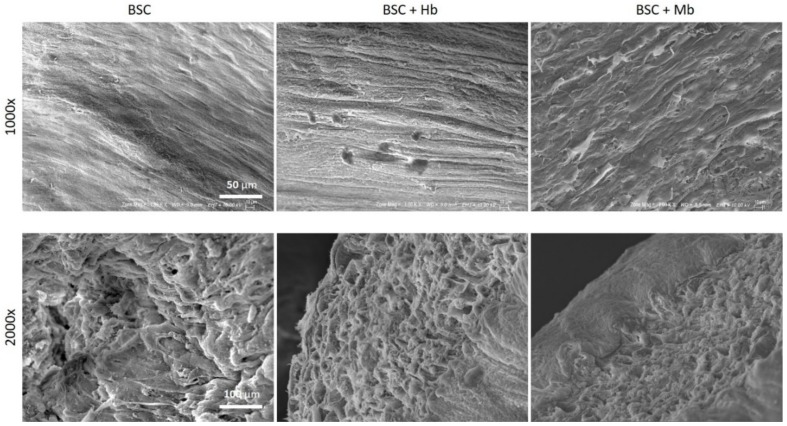
Scanning electron microscopy images of BAMs. BAMs generated from BSC, BSC + Hb, or BSC + Mb (3 mg/mL for both heme proteins) were dehydrated and sputter coated with gold. Images were taken in 1000× magnification (scale bar = 50 µm) in the center of the tissue and 2000× magnification (scale bar = 100 µm) from the side of the tissue.

Cell alignment was also confirmed via SEM imaging (Figure 7). The surface of both treated and untreated BAMs reveals the presence of aligned elongated structures. SEM imaging also demonstrates how BSC hydrogel compaction generates a dense cellular network without visible porous areas.

### 3.5. Biochemical Activity

**Figure 8:**
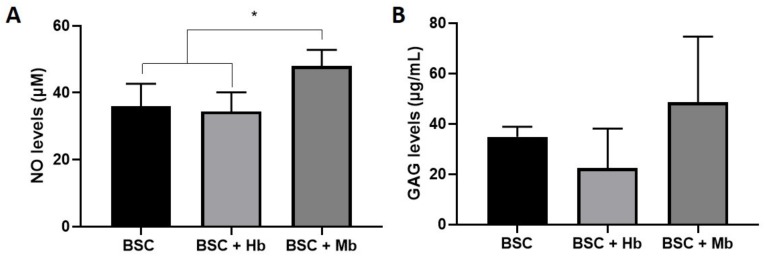
Biochemical analysis of secreted proteins by BAMs. Cell culture media from BAMs generated from BSC, BSC + Hb, or BSC + Mb (3 mg/mL for both heme proteins) was removed after 72 h of incubation and measured for (**A**) Nitric Oxide (*n* = 5) and (**B**) Soluble GAGs (*n* = 6) content. * *p* ≤ 0.05.

As an indicator of overall metabolic activity, we measured the media content of the signaling molecule NO and the secreted ECM molecule GAG (Figure 8). The NO level of the BSC + Mb group was slightly increased compared to the BSCs (*p* = 0.038) and BSC + Hb group (*p* = 0.015). Soluble GAG quantification showed a slight increase in the BSC + Mb group compared with the BSC + Hb group, but the increase was not significant (*p* = 0.07). Taken together, these results indicate a slightly increased metabolic activity in samples incubated with Mb.

### 3.6. Pigment Content and Color of BAMs

**Figure 9:**
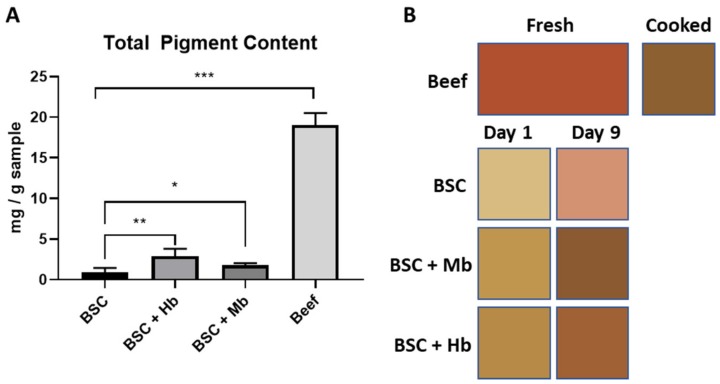
Pigment content and tissue coloration. (**A**) Total pigment content was quantified spectroscopically by homogenization of BAMs generated from BSC, BSC + Hb, or BSC + Mb (3 mg/mL for both heme proteins) and beef (*n* = 6 for all groups) in a sodium phosphate buffer, leading to pigment release into the solution. The amount of pigment was calculated from a standard of Hb and Mb. (**B**) Average L*a*b* values for BSC (n = 6), BSC + Hb (*n* = 5), and BSC + Mb (*n* = 6) and beef (*n* = 9) are displayed. Beef color is presented as fresh or cooked BAM color after incubation of one day or nine days. * *p* ≤ 0.05, ** *p* ≤ 0.01, *** *p* ≤ 0.001.

Total pigment content (both Hb and Mb) of the BAM groups and beef was calculated by absorbance measurement, following homogenization and pigment extraction (Figure 9A). Pigment content in beef was found to be 16.68 ± 1.05 mg/g, which corresponds to the expected heme content in young and mature cattle [35]. BSCs without added heme proteins contained 0.93 ± 0.52 mg/g, and Hb and Mb groups contained 2.89 ± 0.89 mg/g and 1.77 ± 0.25 mg/g respectively. While all BAM groups had significantly lower pigment content compared with beef, the BSC + Hb and the BSC + Mb group had a significantly higher pigment content compared to the BSC group. Taken together, these results show an increased pigment content in groups with added heme proteins, though at a lower level compared to the beef samples.

The color of BAMs was analyzed by digital imaging after one and nine days of incubation. Images were analyzed for L*a*b* values with ImageJ and compared with fresh or cooked beef samples in terms of relative color similarity (ΔE). ΔE shows a high color similarity at low values (identical colors have a ΔE value of zero) and a low color similarity with high values. Results show that heme containing BAMs always had a higher color similarity, both with fresh and cooked beef than the BSC control group (Figure 9B). Interestingly, even though BSC + Hb had the highest pigment content, the highest color similarity after nine days of incubation was found between cooked beef and BSC + Mb (ΔE = 5.4 ± 2.1), followed by BSC + Hb (ΔE = 9.0 ± 0.9); meanwhile, the BSC group had a much lower similarity (ΔE = 24.5 ± 2.4) (Table 1). There was a higher similarity to cooked meat than to fresh meat in all samples. This is a result of the heme redox form (met-form) utilized in this study, which is the redox form present in cooked beef. However, it is expected that different redox forms of heme proteins will have a similar coloration effect on BAMs. With longer incubation times, a decrease in the L* value and an increase in the a* and b* values were observed in all BAM samples, approaching the values of cooked beef. This demonstrates the importance of longer incubation times with heme proteins to facilitate the maturation of tissue and increased coloration. Taken together, these results show that the cultivation of BAMs with heme proteins leads to a higher color similarity with beef than when no heme proteins are applied, which was especially evident for the Mb application.

## 4. Discussion

Cell-based meat is a promising technology to utilize muscle-tissue engineering for food production. In this study, we investigated the role of extracellular added heme proteins Hb and Mb on the development of cell-based meat. We chose these specific heme proteins, as they are either already applied in food products (Hb) or highly abundant in muscle tissue in vivo (Mb). Heme proteins are of interest for in vitro beef production for several reasons. First, the content of heme is responsible for the typical color of meat, and added heme proteins would ideally diminish the necessity of other colorants and resemble the native color of meat more precisely. Second, heme proteins are in part responsible for the slightly metallic taste of beef and can increase the iron content of the final product [30]. Third, Mb has very low expression levels in undifferentiated muscle cells [24,36], but has important functions within differentiated muscle tissue; therefore, incorporation of extracellular Mb might influence biochemical activity and cell proliferation. Our results show that heme proteins have an effect on the proliferation and coloration of the tissue, with Mb showing a more beneficial outcome.

We found that the proliferation of BSCs in 2D was increased in the presence of Mb, while Hb had no, or a slightly negative, effect on cell proliferation. We hypothesize that added Mb increases oxygen transport to mitochondria and subsequent biochemical activity, which could explain the observed increased proliferation potential. To our knowledge, Mb-induced increased proliferation of myosatellite cells was not yet described. However, in other cell types, Mb does not seem to stimulate proliferation. Increased Mb expression in human cancer cells decreases cell proliferation due to interaction with mitochondria [37]. In cell culture, both free Hb and Mb were shown to cause damage to renal cells, starting from concentrations as low as 1 mg/mL [38]. A study observing the effect of Hb on smooth muscle cells showed reduced proliferation potential at concentrations of 100 µM (equivalent to 6.5 mg/mL) due to free radicals, which might explain the slightly decreased proliferation of BSCs upon Hb addition. Hemoglobin was shown to cause damaging effects to tissue due to NO depletion and hemin release [39]. Free heme is known to cause toxicity due to the oxidative potential of free iron, as observed in vivo in certain kidney diseases in humans [38,40]. Thus, the beneficial effect of Mb on myosatellite cell proliferation seems to be cell-specific. Along with other methods, such as genetical engineering of cells [41], the addition of Mb to the cell culture media might therefor be of value for large-scale cell expansion.

After nine days of incubation in a fibrin hydrogel, BAMs had average tissue dimensions similar to previously published results [42,43]; however, some groups have achieved larger constructs with different approaches, such as specialized bioreactors [44], cell-sheet stacking [45], 3D bioprinting [46], or coculture systems with endothelial cells [47]. The size of constructs, however, depends largely on initial cell number, scaffold volume, and cultivation method. Fibrin hydrogel in this study was animal-derived; however, recombinant fibrin formulations were also previously described [48,49]. After eight days of incubation in differentiation media, early myogenesis of BSCs and BSCs + Mb was observed by Troponin T staining. It is possible that, given more time, additional cells may have fused [43]. However, for cell-based meat development, full differentiation of skeletal muscle cells might not be required [50]. SEM imaging and cell alignment quantification furthermore confirmed fibrillar and dense surface structure, comparable to results published by other groups [51,52], and opposed to fibrin hydrogels grown without the presence of cells, which show greater porosity [53], which is beneficial, as fibrillar structures are integral for meat texture. Cell death was observed in deeper layers of the tissue, which is commonly reported, as tissues without blood vessels or perfusable chambers show decreased viability beyond 100–200 µm of the tissue surface, due to an insufficient supply of oxygen and nutrients [54]. Furthermore, high cell-seeding densities, additionally increased by compaction of the hydrogel, are known to cause stress in cell culture, leading to apoptosis. These results underline the importance of development of tissue vascularization or perfusion methods [55]. Biochemical activity appeared to be slightly increased in Mb-treated groups, in terms of secreted NO and GAGs. Skeletal muscle cells produce NO by nitric oxide synthases. NOs are gaseous free radicals that can freely transverse cell membranes and act as signaling molecules, which are associated with muscle repair and regeneration [56,57]. GAGs are part of proteoglycans and have an important function in the binding and storage of growth factors; furthermore, they act as a protein anchorage present in the extracellular matrix to facilitate cell-substrate adhesion. The release of different GAGs, e.g., hyaluronic acid, heparan sulfate, chondroitin sulfate, and chondroitin, by skeletal muscle cells was previously reported [58,59,60]. Increased secretion of these molecules might indicate higher metabolic activity in the muscle tissue.

Globally, a lot of research was performed on color and color stability of meat due to commercial interest in proper meat packaging and display; however, changing the color of bioengineered tissue constructs has evoked little scientific interest to date. To compare coloration between samples, we applied digital imaging methods, which delivered reliable results for meat and other food samples [61,62,63,64]. The color of meat is mainly influenced by the content and redox-form of myoglobin, with oxymyoglobin being bright red and metmyoglobin being brown–red [21]. In this study, we utilized the Hb and Mb in the met-form due to commercial availability, which led to a darker coloration compared to fresh beef, but a very similar color compared with cooked beef, especially when Mb was applied. This is due to oxidation of oxymyoglobin to metmyoglobin during the cooking process in beef. Similar coloration can be expected with different redox forms of heme proteins, and heme proteins can be stabilized with a variety of methods to obtain the oxymyoglobin form [25]. While heme containing BAMs had a more similar color to cooked meat, the color differences were still evident. This could be explained by an insufficient concentration of heme proteins; the cultivation system used, as meat is a multi-cellular tissue and does not solely consist of muscle cells; and by the fibrin hydrogel, which does not properly represent the extracellular matrix of meat. Given these limitations, further optimization of heme protein application for a more sophisticated cell-based meat product might yield a higher color similarity. Taken together, our results show that heme proteins can be applied in cell-based meat production to drive the color of both raw and cooked cell-based meat. Apart from the coloration, heme proteins might also influence the flavor of the product, as currently applied for the plant-based “Impossible Burger”.

Due to availability, heme proteins applied in this study were animal-derived. As a requirement for cell-based meat is the avoidance of animal products, plant-extracted or recombinant heme proteins are of higher interest for actual production processes. Other strategies to increase heme protein content might include a process design that enables higher myoglobin expression by muscle cells. For example, the restriction of iron leads to a 0.5-fold increase in myoglobin content in mice muscle tissue compared to untreated animals [65]. An almost 2-fold increase in myoglobin content in C2C12 cells in 5% lipid supplemented growth media was reported [66]. Up to a 15-fold increase in myoglobin expression in vascular smooth muscle cells was found when treated with nitric oxide [67]. Different groups showed increased myoglobin expression in muscle cells from the addition of 0.5 mM of acetic acid [68] or ursolic acid [69] to the cell culture media. Previous studies also showed that hypoxia leads to increased myoglobin expression [36], and hypoxic cell culture conditions increased BSC proliferation and differentiation [27]. However, muscle cells are one of only a few cell types that maintain cell proliferation under hypoxia, which must be taken into consideration in coculture systems with endothelial cells and adipocytes [70]. These or other changes in process parameters might be of interest to increase myoglobin production by the cells, but difficulties might arise when taking coculture systems, toxicity of certain supplements, or feasible production conditions and/or costs into account.

## 5. Conclusions

We demonstrated that heme proteins, added directly to the cell culture media can influence proliferation of bovine skeletal muscle cells and can lead to a more meat-like coloration of 3D skeletal muscle tissues cultivated in vitro for the generation of cell-based meat. Furthermore, heme protein Mb had preferable attributes compared to Hb as a cell-culture media additive. This study shows the potential of heme proteins to be utilized in the development of cell-based meat.

## Figures and Tables

**Table 1:** Color of BAMs generated from BSC, BSC + Hb, or BSC + Mb (3 mg/mL for both heme proteins) after one or nine days of incubation, compared with fresh and cooked beef samples.

	Beef		BSC		BSC + Hb		BSC + Mb	
	Fresh	Cooked	Day 1	Day 9	Day 1	Day 9	Day 1	Day 9
L*	45.9 ± 3.2	44.5 ± 0.9	76.4 ± 1.5	66.3 ± 3.7	60.9 ± 1.1	47.5 ± 1.7	64.5 ± 1.5	42.8 ± 4.1
a*	37.2 ± 0.6	12.9 ± 0.9	3.5 ± 0.9	20.8 ± 3.3	11.4 ± 1.0	20.9 ± 0.9	8.1 ± 0.7	15.6 ± 1.0
b*	37.7 ± 0.8	33.7 ± 0.7	32.7 ± 0.9	26.8 ± 0.7	42.3 ± 0.6	36.1 ± 0.4	43.1 ± 1.3	31.7 ± 1.4
**ΔE (fresh) ^a^**			45.7 ± 1.5	28.4 ± 4.2	30.1 ± 1.1	16.4 ± 0.9	34.9 ± 1.1	23.0 ± 1.3
**ΔE (cooked)**			33.3 ± 1.6	24.5 ± 2.4	18.6 ± 0.8	9.0 ± 0.9	22.6 ± 1.2	5.4 ± 2.1

^a^ ΔE (fresh) represents color similarity of a given group with fresh beef, and ΔE (cooked) represents color similarity with cooked beef. Values for L* (lightness), a* (red/green spectra), and b* (blue/yellow spectra) are presented. The relative color difference between BAMs and either fresh or cooked beef is expressed as ΔE. A value of ΔE = 0 represents identical colors, while higher values represent a greater difference in color.

## Supplementary Materials

The following are available online at https://www.mdpi.com/2304-8158/8/10/521/s1. S1 File: 3D models of anchor point constructs in different configurations for 24-well plate. Video S1: Time lapse of hydrogel compaction by skeletal muscle cells in the middle of the BAM. Video S2: Time lapse of hydrogel compaction by skeletal muscle cells on the anchor point of the BAM.
