# Supplementary material for: Extracellular Heme Proteins Influence Bovine Myosatellite Cell Proliferation and the Color of Cell-Based Meat
Source: Foods. 2019 Oct 21;8(10):521. doi: 10.3390/foods8100521 (PMC6835221; doi:10.3390/foods8100521)
Supplement: Supplementary file 1 [file foods-08-00521-s001.zip › supplementary/Description of S1 video and S2 video.docx]

**Description for S1 Video and S2 Video:**

Time Lapse Video showing formation of muscle construct in fibrin hydrogel along two anchor points. BSCs were added to a fibrin hydrogel and incubated with controlled temperature and CO_2_ inside a KEYENCE microscope. Images from the center of the construct (Video 1) or the anchor point (Video 2) were taken every 15 min for a total time of 12 h in brightfield channel with z-Stack. Scale bar represents 200 µm.
